# Unidirectional valved patches for closure of septal defects in patients with severe pulmonary hypertension

**DOI:** 10.4103/0974-2069.43876

**Published:** 2008

**Authors:** Sachin Talwar, Shiv Kumar Choudhary, Anita Saxena, Shyam Sunder Kothari, Rajnish Juneja, Balram Airan

**Affiliations:** Cardiothoracic Centre, All India Institute of Medical Sciences, New Delhi, India

**Keywords:** Congenital heart disease, pulmonary hypertension, septal defects

## Abstract

Pulmonary hypertension due to delay in presentation, diagnosis, referral, and surgery for septal defects is not uncommon in the developing world and translates into high morbidity and mortality following open heart surgery to close these defects. Leaving a small atrial communication may not always be effective. Extracorporeal membrane oxygenation and inhaled nitric oxide therapy in the immediate postoperative phase may not be available or may not be financially feasible in many institutions which are economically challenged. Unidirectional valved patch is emerging as a new and effective solution to this problem and promises to make at least the immediate postoperative results more predictable in this subset of patients.

## INTRODUCTION

In developing countries, it is not uncommon to encounter patients with ventricular and atrial septal defects who present beyond infancy.[1,2] These patients often have severe pulmonary arterial hypertension (PAH) with a high pulmonary vascular resistance with reversed or bidirectional shunt through a septal defect. Many of these patients are considered to have “borderline operability”. The operations to correct these defects are conducted using cardiopulmonary bypass (CPB) that is associated with a systemic inflammatory response causing release of vasoactive substances (thromboxane A2 and catecholamines), which results in pulmonary vasoconstriction and acute pulmonary hypertension.[3–6] The resulting pulmonary hypertensive crisis, acute congestive heart failure, and acute respiratory failure are the principal causes of increased morbidity with mortality ranging from 22.7–50%.[7–15] To counter these problems, patients require high doses of pharmacologic agents such as phosphodiesterase inhibitors like sildenafil or inhalational nitric oxide to achieve hemodynamic stability in the immediate postoperative phase. In extreme cases, difficulty may be encountered in separating these patients from cardiopulmonary bypass and extracorporeal membrane oxygenation (ECMO) support may be required. In industrialized nations, such patients are potential candidates for lung or heart-lung transplantation or aggressive pulmonary vasodilator therapy following closure of septal defects. All currently available pulmonary vasodilators with the exception of sildenafil are very expensive and have limited efficacy. Some of the available agents such as prostacycline and inhaled nitric oxide are difficult to administer on a long-term basis. Elastase inhibitors and gene transfer therapy are still experimental even in the developed world.[16] Despite these strategies the “long-term” survival remains unchanged.[7–11] Therefore, these expensive measures are logistically impractical and are a drain on the meager health resources in developing nations.

A common surgical strategy to improve outcome in these patients consists of leaving behind a small interatrial communication to provide a pop-off during periods of elevated right-sided pressures when the resultant right-to-left shunt through the interatrial communication prevents acute right ventricular failure at the cost of systemic desaturation. The degree of shunting through this communication is unpredictable, and rarely some of these may require percutaneous intervention to close this communication if the pulmonary artery pressures subside and a left-to-right shunt ensues. A fenestration in the patch used to close the defect with a valved mechanism can potentially serve as a pop-off in one direction at the level of the previous shunt (atrial or ventricular septal defect). When the left-sided pressures exceed right-sided pressures, the valve closes and prevents a left-to-right shunt.

### Principles

**Figure 1:**
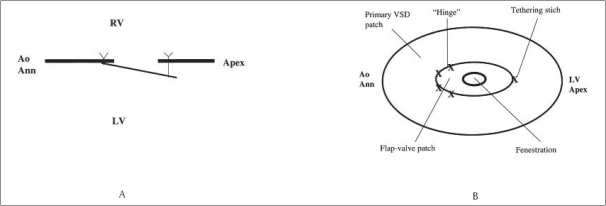
(A) Frontal view of flap valve ventricular septal defect (VSD) patch. (Ao Ann, aortic annulus; LV, eft ventricle); (B) Lateral view of flap valve ventricular septal defect (VSD) patch. (Ao Ann = aortic annulus; LV = left ventricle; RV = right ventricle.) Reproduced with permission from Novick WM, Gurbuz AT, Watson DC, Lazorhysynets VV, Perepeka AN, Malcic I, et al. Double patch closure of ventricular septal defect with increased pulmonary vascular resistance, Ann Thorac Surg 1998; 66:1533–1538. Copyright Society of Thoracic Surgeons and Elsevier

A variety of techniques have been utilized for creating a unidirectional valved patch (UVP).[17–21] The principles of all these techniques essentially remain the same. These patches are designed to function like the fossa ovalis of the atrial septum. During periods of acute elevation of pulmonary artery pressure, opening of the valve allows the blood to flow from right to left. This right-to-left shunt prevents acute right ventricular failure from refractory pulmonary artery hypertension and helps in maintaining adequate cardiac output, thereby, reducing the risk of early postoperative death [Figure 1]. When pulmonary artery pressure gradually falls after operation and pressure gradients between the right and left sides of the circulation normalize, the unidirectional valve closes and prevents a significant left-to-right shunt [Figure 1].

### Evolution and Techniques

**Figure 2:**
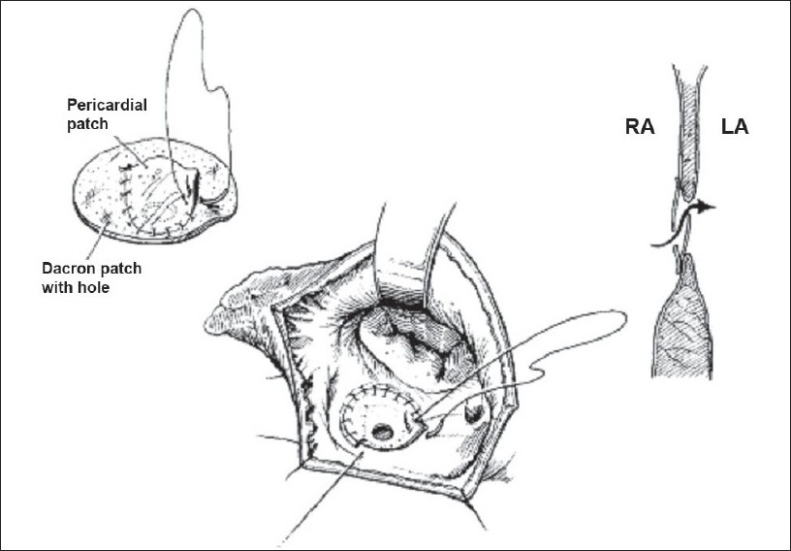
Zhou's technique. Three sides of the pericardial patch are attached and one side is open to function as a valve. The pericardial valve flap is placed on the left side of the defect. This allows the blood to flow from the right atrium (RA) to the left atrium (LA) or across the ventricular septum when used to close the ventricular septal defect. (Reproduced with permission from Zhou Q, Lai Y, Wei H, Song R, Wu Y, Zhang H. Uidirectional valve patch for repair of cardiac septal defects with pulmonary hypertension. Ann Thorac Surg 1995;60:1245–9, Copyright Society of Thoracic Surgeons)

In 1995, Zhou *et al*.,[17] first described UVP for closure of ventricular septal defects (VSD) in patients with severe PAH [Figure 2]. The UVP was constructed from a Dacron patch approximately as large as the size of the defect to be closed. A fenestration about 0.5–1.0 cm in diameter was made in the patch, somewhat off the center. A piece of quadrangular pericardium was attached by suture to the surface of the Dacron patch. The three edges of pericardium were continuously sutured and one edge was left unattached. This unattached edge of pericardium was tailored to provide a small opening, not exceeding 0.5 cm, and appropriate tension was maintained on the pericardial edge so that the valve mechanism is not loose and incompetent. Also, excessive tension was avoided and complete attachment of the pericardium to the Dacron patch was avoided to prevent the valve from losing its effectiveness. The pericardial flap valve was placed on the left (systemic) side of the defect.

**Figure 3:**
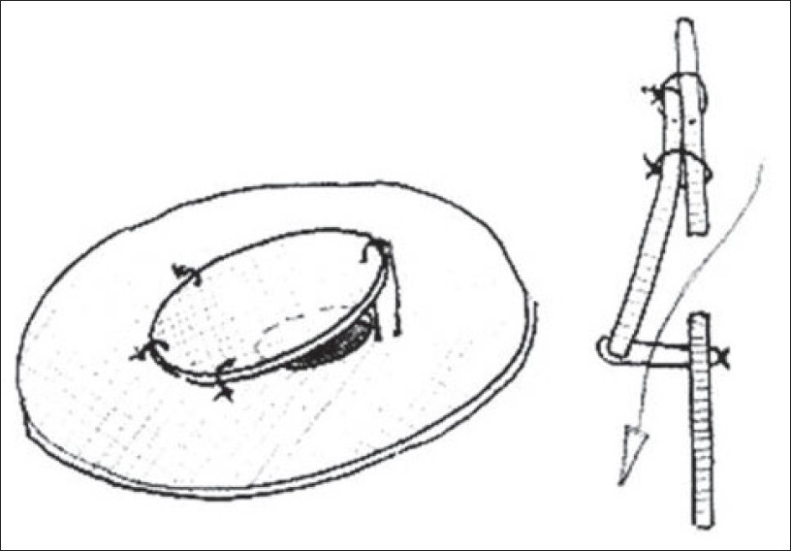
Illustration of double flap valve patch in profile with open valve. (Reproduced with permission from Novick WM, Sandoval N, Lazorhysynets VV, Castillo V, Baskevitch A, Mo X, et al. Flap valve double patch closure of ventricular septal defects in children with increased pulmonary vascular resistance. Ann Thorac Surg 2005;79:21–8. Copyright Society of Thoracic Surgeons and Elsevier)

Novick *et al*,[18] introduced the first modification [Figures 1 and 3]. They constructed the UVP from sauvage Dacron (C.R. Bard, Haverhill, MA) and Gore-Tex (W.L. Gore and Assoc, Flagstaff, AZ) cardiovascular patches. After tailoring the patch for primary closure of the VSD, a fenestration was made in the central region of the patch. The weight of the child was used to decide the size of the fenestration. For children weighing 15 kg or less, a 4-mm fenestration was made; for those weighing between 15 and 20 kg, a 5-mm fenestration was made; and for those weighing more than 20 kg, a 6-mm fenestration was made. A separate flap valve patch measuring at least 4 mm greater than the diameter of the fenestration was then constructed. This flap valve patch was sewn to the superior aspect of the fenestration on the left ventricular side of the VSD patch. Interrupted sutures were placed along one-third of the superior rim of the flap valve patch to anchor it to the VSD closure patch. A separate tethering suture was placed inferiorly and tied loosely to approximate the size of the fenestration. The constructed patch was finally sewn into place to close the VSD. The important point was that the fenestration was made in the center of the patch for all defects except atrioventricular septal defects (AVSD). In patients with AVSD, the fenestration was placed in the lower third of the patch to prevent the flap valve from interfering with left atrioventricular valve excursion. In 2005, they published a modified technique and proposed a fenestration size that was half of the expected aortic annulus diameter for each child.[18] Following this, a separate flap patch at least 4 mm larger than the fenestration was constructed and sewn onto the superior margin of the fenestration along one-third of the circumference. A separate tethering stitch was placed at the inferior apex of the flap valve and tied loosely over a Hegar dilator that was the same size as the fenestration. This tethering stitch length approximated the diameter of the fenestration. The VSD patch was sewn into place orienting the patch in such a way that the flap valve was placed on the left ventricular (LV) side with the flap opening towards the LV apex to avoid subaortic obstruction. Novick *et al*.,[18,19] recommend the use of this technique to improve the quality of life even for those patients who are declared inoperable and in whom alternative therapies for irreversible PAH, like heart-lung transplantation are not an option.

**Figure 4:**
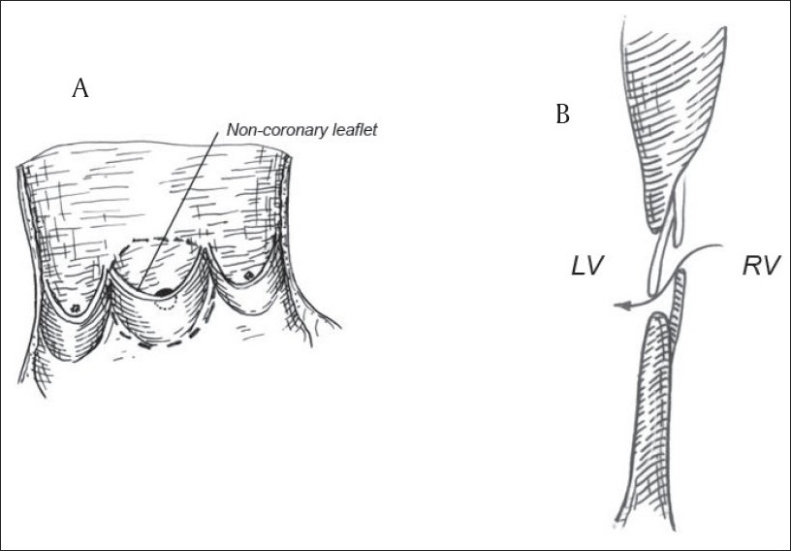
(A) Double flap valve patch with aortic homograft. Noncoronary leaflet and anterior mitral leaflet are used to form monovalved patch which is fenestrated above the nadir of noncoronary leaflet; (B) Open valve:lateral view of flap valve and ventricular septal defect patch. (LV, left ventricle; RV, right ventricle.) (Reproduced with permission from Zhang B, Wu S, Liang J, Zhang G, Jiang G, Zhou M, *et al*, Unidirectional monovalve homologous aortic patch for repair of ventricular septal defect with pulmonary hypertension. Ann Thorac Surg 2007;83:2176–81.Copyright Society of Thoracic Surgeons and Elsevier)

In 2007, Zhang *et al*, proposed the use of aortic homograft along with the attached mitral leaflet to construct the UVP [Figure 4].[20] The aortic valve homograft was opened longitudinally at the commissure between the right and left coronary leaflet. The right and left coronary leaflet were removed but the noncoronary leaflet and anterior mitral leaflet were preserved to form a large aortic patch with the monovalve. A 4–8 mm fenestration was made above the nadir of the noncoronary leaflet. For patients with body surface area (BSA) less than 1 m^2^ and with a preoperative saturation of greater than 91%, a 4-mm fenestration was made; a 6-mm fenestration was made if the patient's BSA was less than 1 m^2^ and preoperative SaO_2_ was less than 91% or the patient's BSA was larger than 1 m^2^ and preoperative SaO_2_ higher than 91%; and an 8-mm fenestration was made if the patient's BSA was larger than 1 m^2^ and preoperative SaO_2_ was less than 91%. The patch was sutured to close the VSD with the noncoronary leaflet facing the left ventricular side and its free edge toward left ventricular outflow tract.

**Figure 5:**
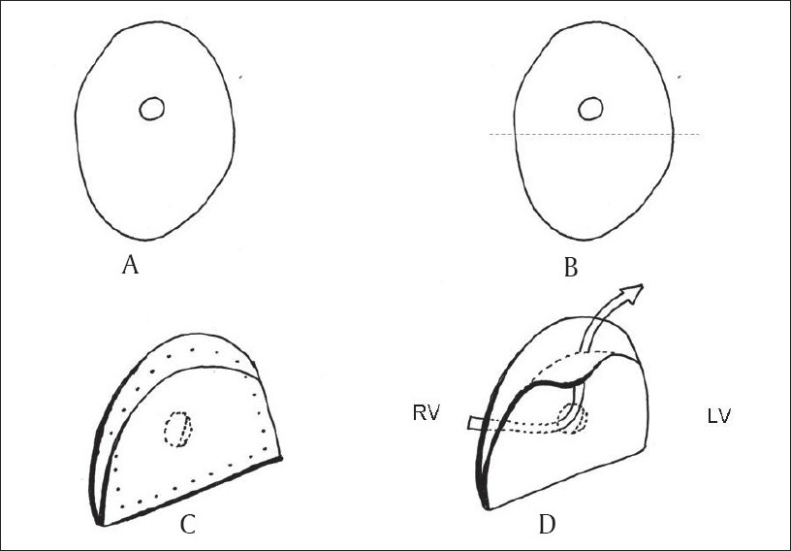
AIIMS technique of unidirectional valved patch. (A) appropriate size Dacron patch is selected and a fenestrated (B) patch is folded upon itself at the dotted line (C) the patch is sutured (outer dots) to close the defect (D) mechanism of right to left shunting through the patch in-situ (arrow). Suture line has been removed for clarity. Redrawn with permission from Choudhary SK, Talwar S, Airan BA. A simple technique of unidirectional valved patch for closure of septal defects. J Thorac Cardiovasc Surg 2007;134:1357–8. Copyright American association for thoracic surgery and Elsevier)

We have developed a simplified technique at our institution to address the limitations posed by other techniques. This technique is described in detail elsewhere.[21] After inspecting the defect, a patch of knitted polyester fabric (Impra Inc, Tempe, AZ, US), which is the same width as the size of the defect but is approximately one and half times longer than the desired length is selected and a 4-mm fenestration is made into it. The patch is then folded upon itself to achieve adequate dimensions for closure of the septal defect. The flap so created by the folding of the patch covers the fenestration and provides a valved mechanism [Figure 5]. This patch is sutured to the edges of the septal defect so that the flap lies toward the left ventricular side in patients with VSD and toward the left atrial side in patients with atrial septal defect (ASD). Care should be taken to orient the flap to open downwards toward the LV apex so that it does not produce left ventricular outflow tract obstruction during systole.

### Postoperative management

It has been our practice to start dopamine 5–10 *µ*g/kg/min, nitroglycerin or nitroprusside 0.5–2 *µ*g/kg/min, or the phosphodiesterase inhibitor milrinone prior to discontinuation of CPB. Some advocate placing transthoracic pulmonary artery catheters in all patients to monitor pulmonary artery pressures in the postoperative phase. Intraoperative transesophageal echocardiography and postoperative transthoracic echocardiography is used to assess the result of the operation and the degree of shunting across the fenestration. After termination of CPB, moderate hypocarbia is commonly employed in the operating room. Once the patients are fully awake and hemodynamically stable, they are extubated. Upon resuming oral intake, oral angiotensin converting enzyme inhibitor or phenoxybenzamine is started and the inotropes are weaned off. Sildenafil may be administered depending upon the institutional protocol. Anticoagulants/aspirin are not administered.

After discharge from the hospital, follow-up consists of clinical examination measurement of SpO_2_ using pulse oximetry and trans-thoracic echocardiography at regular intervals to estimate the pulmonary artery (PA) pressure, assess the right ventricular function and demonstrate the degree of right-to-left shunting. Cardiac catheterization is usually not indiacted.

## RESULTS

The results obtained with the use of UVP by various groups are summarized in [Table 1]. Two deaths in Zhou's experience[17] were during the learning curve. One patient died due to acute right ventricular failure and another due to respiratory failure. In the initial 18 patients reported by Novick,[18] there were no early deaths and one late death occurred while the child was awaiting detailed evaluation for suspected pulmonary stenosis. In Novick's later experience[19] spread over more than 10 centers around the world, there were seven early deaths. These were due to technical errors (n = 3), pneumonia (n = 2), arrhythmia (n = 1) and hemorrhage following removal of PA line (n = 1). No cause was specified for the seven late deaths. However, a trivial right-to-left shunt was still present in four survivors at follow-up (time not specified). Postoperative echocardiographic studies revealed persistent PAH in 25 patients and normal pressures in 17 patients. In Zhang's experience of 27 patients,[20] there were two early deaths due to persistent PAH but there were no late deaths. In our own experience with 21 patients,[22] there were no early or late deaths, but our maximum follow-up was only for a period of 18 months. The valved mechanism was always found to work effectively in patients with suprasystemic right ventricular pressures, who needed a pop-off and this was observed on postoperative transthoracic echocardiograms. No patient had a left-to-right shunt when the RV pressure was subsystemic.

**Table 1:** Summary of results obtained with the use of unidirectional valved patch

Author	Year	No	Age (years)	Pre-op SpO_2_ (%)	PA pressures (mm Hg)	PVRI (Wood units)	Qp:Qs	PASP/AOSP	Early deaths	Postop SpO_2_ (%)	Early R-L shunt	Postop PA Pressures (mm Hg)	Follow-up in yrs (mean)	Late deaths	No: with R - L shunt at last follow-up
Zhou[17]	1995	24	5–28 (15.8)	82–97 (87 ± 4)	80 ± 12	8–32 (16 ± 7)	0.5–2 (1.2 ± 0.5)	1–1.3 (1.1 ± 0.1)	2(8.3%)	96 ± 1	12	56 ± 18	0.3–3 (1.1)	Nil	Nil
Novick[18]	1998	18	1.5–15 (5.7 ± 3.9)	89 ± 5	105 ± 16	11.4 ± 4.1	1.4 ± 0.41:1	NA	Nil	96 ± 2	**	42 ± 14	Details NA	1	Details NA
Novick[19]	2005	91	0.5–17 (4 ± 3.1)	90 ± 4	NA	10.5 ± 4.9	1.6 ± 0.8	0.96 ± 0.12	7(7.7%)	±	8	NA	0.1 - 7.6	7 / 78# (8.9%)	4
Zhang[20]	2007	27	6–31 (15 ± 5.6)	89 ± 1	81 ± 12	(2.4 ± 2.5)	0.5–2 (1.2 ± 0.5)	1.05 ± 0.1	2 (7.4%)	95 ± 2	10	68 ± 15	0.5-10 (4.1 ± 1.3)	Nil	2
AIIMS*	2007	21	2–37 (5 ± 1.9)	91 ± 3.4	88 ± 10	10.6 ± 4.3	1.6 ± 0.6	NA	Nil	±	9	NA	1–-1.5	Nil	1

* : Unpublished data.[22]

** : Fluctuations were observed. Number not specified

# : Follow-up data were available only for 78 patients, Figures in parentheses are mean values unless indicated otherwise, PA: pulmonary artery, PVRI: pulmonary vascular resistance, PASP: pulmonary artery systolic pressure, AOSP: aorta systolic pressure, NA: Not available

### Merits and limitations

All the described techniques have their advantages and disadvantages. It is difficult to compare the results of various techniques because of small numbers and heterogeneity of the patient population. These series have included a wide range of patients with diagnosis such as VSD, ASD, AVSD, double outlet right ventricle, total anomalous pulmonary venous drainage, etc. The overall results observed in these series have been gratifying. However, all the earlier techniques have been found to be time consuming. The UVP in these techniques is always constructed after cardioplegic arrest and assessment of the defect as fashioning the patch prior to this may result in improper sizing of the patch. Also, two patches are often required to construct the UVP, a procedure which adds to the ischemia time. In the original description by Novick, one patient died due to left ventricular outflow tract obstruction due to the movement of the patch necessitating a further modification whereby the valve was placed toward the apex of the left ventricle. The technique described by Zhang[20] is innovative, but suffers from the need for a homograft which are costly and in limited supply. To overcome these disadvantages, we devised a new technique[21] that requires the use of only one patch and less than half a minute to fashion. It is safe, effective, and easily reproducible in our early experience.[22] More detailed results with long-term follow up of our technique are awaited.

Unfortunately, in the absence of a randomized study with controls, it is not possible to conclusively demonstrate that a UVP can actually reduce the mortality and improve survival. The magnitude of right-to-left shunt has not been quantified in any of these studies. Prospective studies with a detailed evaluation and description of the patients’ resting PO_2_ before and after surgery, and response to exercise with demonstration of the direction of shunt through the patch using contrast echocardiography may potentially answer questions about the long-tem usefulness and integrity of the valve mechanism. Cardiac catheterization with detailed hemodynamic evaluation can be performed if the patients consent to undergo this procedure.

## CONCLUSIONS

The UVP is designed only to tide over the period of hemodynamic instability in patients with septal defects and severe PAH and to provide a safe closure of the septal defects in patients with "border-line operability". Given the present day indications, this approach helps to salvage these patients in the short to mid-term but the long-term effects of this technique on the natural history of patients with long standing severe PAH are unknown. We have no data to support the use of this technique in patients with established Eisenmenger's syndrome.

## References

[1] World health organization (1985). Health manpower requirements for the achievement of health for all by the year 2000. WHO: Technical Report Series.

[2] Saxena A (2005). Congenital heart disease in India: A status report. Indian J Pediatr.

[3] Montalescot G, Lowenstein E, Ogletree ML, Greene EM, Robinson DR, Hartl K (1990). Throm- boxane receptor blockade prevents pulmonary hypertension induced by heparin-protamine reactions in awake sheep. Circulation.

[4] Komai H, Yamamoto F, Tanaka K, Yagihara T, Kawashima Y (1994). Prevention of lung injury during open heart operations for congenital heart defects. Ann Thorac Surg.

[5] Heerdt PM, Weiss CI (1990). Prostaglandin E1 and intrapulmonary shunt in cardiac surgical patients with pulmonary hypertension. Ann Thorac Surg.

[6] Weesner KM (1991). Hemodynamic effects of prostaglandin E1 in patients with congenital heart disease and pulmonary hypertension. Cathet Cardiovasc Diagn.

[7] Arciniegas E, Glen WWL (1983). Ventricular septal defect. Thoracic and cardiovascular surgery.

[8] Steele PM, Fuster V, Cohen M, Ritter DG, McGoon DC (1987). Isolated atrial septal defect with pulmonary vascular ob- structive disease: Long-term follow-up and prediction of outcome after surgical correction. Circulation.

[9] Hallman GL, Cooley DA, Wolfe RR, McNamara DG (1964). Surgical treatment of ventricular septal defect associated with pulmonary hypertension. J Thorac Cardiovasc Surg.

[10] Cartmail TB, DuShane JW, McGoon DC, Kirklin JW (1966). Results of repair of ventricular septal defect. J Thorac Cardiovasc Surg.

[11] Friedli B, Kidd BS, Mustard WT, Keith JD (1974). Ventricular septal defect with increased pulmonary vascular resistance. Am J Cardiol.

[12] John S, Korula R, Jairaj PS, Muralidharan S, Ravikumar E, Babuthaman C (1983). Results of surgical treatment of ventricular septal defect with pulmonary hypertension. Thorax.

[13] Guo JQ, Xue GX, Zhu XD, Yue XH, Hu BZ, Zhi QH (1983). Surgical treatment of congenital ventricular septal defect: A 21 years experience in 1187 patients. Chin Med J (Engl).

[14] De Souza CA, Spyt TJ (1992). Release of vasoactive substance during cardiopulmonary bypass. Ann Thorac Surg.

[15] Komai H, Yamamoto F, Tanaka K, Murashita T, Shibata T, Sakai H (1993). Increased lung injury in pulmonary hypertensive patients during open heart operations. Ann Thorac Surg.

[16] Wessel DL (2001). Current and future strategies in the treatment of childhood pulmonary hypertension. Progr Pediatr Cardiol.

[17] Zhou Q, Lai Y, Wei H, Song R, Wu Y, Zhang H (1995). Uidirectional valve patch for repair of cardiac septal defects with pulmonary hypertension. Ann Thorac Surg.

[18] Novick WM, Gurbuz AT, Watson DC, Lazorishinets VV, Perepeka AN, Malcic I (1998). Double patch closure of ventricular septal defect with increased pulmonary vascular resistance. Ann Thorac Surg.

[19] Novick WM, Sandoval N, Lazorhysynets VV, Castillo V, Baskevitch A, Mo X (2005). Flap valve double patch closure of ventricular septal defects in children with increased pulmonary vascular resistance. Ann Thorac Surg.

[20] Zhang B, Wu S, Liang J, Zhang G, Jiang G, Zhou M (2007). Unidirectional monovalve homologous aortic patch for repair of ventricular septal defect with pulmonary hypertension. Ann Thorac Surg.

[21] Choudhary SK, Talwar S, Airan B (2007). A simple technique of unidirectional valved patch for closure of septal defects. J Thorac Cardiovasc Surg.

[22] Talwar S, Choudhary SK, Airan B, Saxena A, Kothari SS, Juneja R Unidirectional valved patch for closure of septal defects in patients with severe pulmonary hypertension.

